# A six question screen to facilitate primary cardiovascular disease prevention

**DOI:** 10.1186/s12872-015-0131-0

**Published:** 2015-10-30

**Authors:** Niels V. van der Hoeven, Maurice A. J. Niessen, Erik S. G. Stroes, Lex Burdorf, Roderik A. Kraaijenhagen, Bert-Jan H. van den Born

**Affiliations:** Departments of Internal and Vascular Medicine, Academic Medical Center of the University of Amsterdam, Meibergdreef 9, 1105AZ Amsterdam, The Netherlands; NIPED Research Foundation, Amsterdam, The Netherlands; Department of Public Health, Erasmus MC, Rotterdam, The Netherlands

**Keywords:** Cardiovascular disease, Prevention, Risk prediction, Risk assessment, Risk management, SCORE

## Abstract

**Background:**

European guidelines on primary prevention of cardiovascular disease (CVD) recommend the SCORE risk charts for determining CVD risk, which include blood pressure and serum cholesterol as risk parameters. To facilitate cost-effective large-scale screening, we aimed to construct a risk score with ‘non-invasive’ parameters as a first screening step to identify persons at increased CVD risk requiring further risk assessment.

**Methods:**

We used data of Dutch employees from 25 organisations participating in a health risk assessment between August 2007 and January 2013. Backward multivariate logistic regression analysis was employed to select non-invasive, independent predictors of high CVD risk, defined as the 10-year risk of fatal CVD of ≥5 % based on the SCORE formula. The total CVD risk score was calculated as the summed coefficients of the retained variables.

**Results:**

Data of 6189 male participants was used for the development and validation of the risk score. Age, tobacco use, history of hypertension, alcohol consumption, BMI, and waist circumference were independent predictors of high CVD risk. Ten-fold cross-validation resulted in an area under the curve of 0.95 (SE 0.01, 95 % confidence interval 0.94–0.96). A cut-off score ≥45 on the CVD risk score yielded a sensitivity of 0.93, and a specificity of 0.85.

**Conclusions:**

We developed a simple, non-invasive risk score that accurately identifies persons at increased CVD risk according to the SCORE formula in a population of working men. The risk score enables a stepwise approach in large screening programmes, strongly reducing the number of persons that require full risk estimation including blood pressure and cholesterol measures.

**Electronic supplementary material:**

The online version of this article (doi:10.1186/s12872-015-0131-0) contains supplementary material, which is available to authorized users.

## Background

Cardiovascular disease (CVD) is the major cause of premature death in Europe [1, 2]. Despite the identification of modifiable risk factors such as smoking, sedentary lifestyle, blood pressure (BP) and dyslipidemia [3], prevention of CVD remains challenging. A complicating factor is that treatable cardiovascular risk factors can be silently present for many years before detection by routine check-up or the occurrence of a cardiovascular event.

Early detection of individuals at high CVD risk is the cornerstone of primary prevention. For estimation of CVD risk current guidelines from the joint task force of the European Association for Cardiovascular Prevention and Rehabilitation [4] recommend the use of the SCORE (Systemic COronary Risk Evaluation) risk estimation [5]. Based on age, gender, smoking status, cholesterol and BP an estimation of the 10-year risk of dying from CVD can be calculated, or derived from a risk chart. The risk estimation is used to offer the individual tailored health advice, including behavioural strategies to improve lifestyle and pharmacological interventions aimed at reducing BP and cholesterol. For practical reasons it is currently recommended to assess cardiovascular risk in all men over 40 and women over 50 years of age or post-menopausal without CVD [6]. However, population-wide screening of all persons meeting these criteria is a very costly and time-consuming effort, making it an unattractive approach for everyday practice [7]. The use of a simple, non-invasive risk score based on current guideline recommendations as a first step in the screening process might overcome these barriers and facilitate large scale CVD screening. Such a risk score can be used to select individuals who are likely to be at high CVD risk after performing a full SCORE risk estimation including BP measurements and blood sampling, thereby significantly reducing the costs and labour-intensiveness for CVD screening. Risk scores have been developed to identify patients at increased risk for diabetes [8–10], kidney disease [11], or a combination of cardiometabolic endpoints [12], but not for CVD risk estimation according to the SCORE equation.

In the present study, our aim was therefore to facilitate cardiovascular screening in primary care according to current European guidelines by developing a CVD risk score using simple, non-invasive parameters to identify persons at high CVD risk according to SCORE risk estimation. To this end, we used the data of a large web-based health risk assessment (HRA) carried out in the Netherlands.

## Methods

### Participants

The current study was performed as part of a worksite HRA implemented in Dutch organisations between August 2007 and January 2013. Study participants were employees aged 40–70 years that completed the HRA within this timeframe. Pregnant woman were excluded from enrolling in the HRA. Because the prediction tool was aimed at identifying previously undetected persons at high CVD risk, employees with established CVD or on current treatment for hypertension, hypercholesterolemia, diabetes or chronic kidney disease were excluded from analysis. Informed consent was obtained from all participants prior to the study in accordance with the requirements for identifiable data collection in the Dutch Code of Conduct for Observational Research (http://www.federa.org/sites/default/files/digital_version_first_part_code_of_conduct_in_uk_2011_12092012.pdf).

### Health risk assessment

Details of the HRA have been described previously [13]. In brief, invitations to participate in the HRA were sent by the human resources department, management, or the safety, health, and welfare services of the organizations involved. The invitation included a description of the HRA and informed employees that participation was voluntary and free of charge, that all personal data would be treated confidentially, and that no individual results would be shared with their employer or any other party.

Attendees completed a web-based electronic health questionnaire which included ~100 questions covering socio-demographics, personal health history, family risk and the behavioural domain. This was followed by biometric measurements including length, weight and waist circumference conducted at the worksite by certified staff. Two BP measurements were taken after 5 min of relaxation with a validated oscillometric device. If both systolic measurements were below 140 mmHg, the mean was used for analyses. When at least one of the systolic readings was ≥140 mmHg, participants were instructed to relax for another 30 min in a secluded area after which a third BP measurement was taken. The mean of all three measurements was then used for analyses. At the same visit blood samples were collected for determination of total cholesterol, HDL-cholesterol, LDL-cholesterol, triglycerides, glucose, creatinine, and HbA1C. Creatinine was used to calculate the estimated glomerular filtration rate according to the CKD-EPI formula [14], and is expressed in mL/min per 1.73 m^2^. A urine sample was detected to assess the albumin/creatinine ratio (ACR). Increased ACR was defined as ≥3.5 for male, and ≥2.5 for female persons. A personalised web-based health report and health plan was automatically generated when all health data were collected after which the HRA was completed.

### Assessment of CVD mortality risk

CVD mortality risk was assessed according to the SCORE risk estimation. The SCORE risk estimation predicts the 10-year risk of dying from CVD based on data of 12 large European cohort studies [5]. Variables in the SCORE risk estimation include age, gender, current smoking status, systolic BP (mmHg), and total cholesterol (mmol/L) or the total cholesterol/HDL ratio. In the current study we used total cholesterol to calculate SCORE. Because current guidelines recommend to offer health advice and consider medical treatment in persons with a predicted 10-year CVD mortality risk of ≥5 %, this threshold was used for our primary analysis [1]. The Netherlands constitutes a low risk region in terms of CVD mortality, therefore the SCORE risk formula for low risk regions was used [5].

### Potential predictor variables

For the development of the screening tool, non-invasively assessed variables with a possible association with CVD risk were selected from the HRA. This selection was independently carried out by two physicians (NvdH and DES). Disagreement between the two physicians was resolved through discussion moderated by a specialist in cardiovascular medicine (BJvdB), who gave the decisive vote. A total of 23 non-invasively assessed variables were selected as potential predictors for CVD.

Date of birth, gender, marital status, education and ethnicity were selected from questions related to socio-economic status. For marital status, participants selected one of six categories. Education level was defined as the highest education completed and was stratified in three categories, low (lower general secondary and lower vocational), middle (higher general secondary, pre-university and intermediate vocational), and high (higher vocational, university and doctorate). Ethnicity was defined according to parental background. As the majority of participants were of European descent, the non-European descent answer categories were merged into “other”.

Self-rated health was assessed, as previously described, by the question “How do you rate your health in general?”, and categorised in strata ranging from poor to very good [15, 16]. Frequency of tobacco use was stratified in none, occasionally, weekly, or daily. Alcohol consumption was reported according to the Dutch Municipal Health Service questionnaire, which records the number of consumed alcohol units per week using a semi-quantitative scale. Low vegetable and fruit intake was defined as an average consumption of less than 3 tablespoons of vegetables or 2 pieces of fruit per day. Fat intake was estimated based on the daily consumption of butter, margarine, cheese and other sandwich fillings. Low fish consumption was defined as less than 1 fish meal per week. In accordance with the INTERHEART study [17], two items relating to stress at home and stress at work were combined into a general stress scale and graded as follows: 1) never experienced stress; 2) experienced some periods at home or work; 3) experienced several periods at home or work; 4) experienced permanent stress at home or work. Physical activity was self-assessed by one item derived from the Dutch version of the International Physical Activity Questionnaire (IPAQ) [18]. Participants entered the number of weekdays on which they spent at least 30 min on moderate to vigorous physical activity. Moderate physical activity includes activity that increases respiratory rate, but still allows a person to talk, such as taking a firm walk, swimming, or gardening. Vigorous physical activity includes activity which increases respiratory rate to a level at which a person cannot easily talk anymore, such as intensive exercise, running, or cycling with a speed of ≥17 km/h. Distress was self-assessed with the validated Dutch version of the Extended Kessler distress scale (EK-10) [19, 20], ranging from 10 (no distress) to 50 (severe distress) with a cut-off score of ≥20. First degree family history of CVD (diagnosed before age 60), diabetes mellitus and hypertension was self-reported. History of diabetes mellitus, hypertension, hypercholesterolemia, renal insufficiency was assessed by asking if participants were ever treated for diabetes, blood pressure, high cholesterol or renal insufficiency. Subsequently, persons were asked whether they were still using medication for the selected condition(s). Mental health problems were considered present if participants received treatment for a mental health disorder, such as depression or anxiety. Self-reported length and weight were used to calculate body mass index (BMI) which was categorised into normal (BMI <25 kg/m^2^), overweight (BMI ≥25 and <30 kg/m^2^) and obese (BMI ≥30 kg/m^2^). A waist circumference of ≥94 cm for men and ≥80 cm for women was considered indicative of the presence of visceral adiposity.

### Statistical analysis

Descriptive statistics were used to present the baseline characteristics of the study population. Univariate logistic regression was performed to determine the single effects of potential predictors on a CVD mortality risk of ≥5 %. Variables with a *p*-value <0.10 in the univariate logistic models were entered in the multivariate model. After stepwise backward elimination of predictors the final model included variables with a *p*-value of <0.05. Continuous variables were categorised to simplify its use. The total CVD risk score was calculated as the summed coefficients of the retained variables. Area under the curve (AUC) analysis was used as a measure of overall test performance. An AUC of ≥0.80 is considered indicative of a useful screening instrument [21]. Sensitivity, specificity, positive predictive value, negative predictive value, positive likelihood ratio and negative likelihood ratio were calculated at various cut-off values on the total CVD risk score. All analyses were performed using IBM SPSS version 19 (SPSS Inc., Chicago, Illinois, USA). The data was openly available to the authors during the study time. All interested parties can obtain the data needed to replicate the findings upon request. Parties can contact Dr R.A. Kraaijenhagen (r.a.kraaijenhagen@niped.nl) for these requests.

### Internal validation

K-fold cross-validation was performed in which 10 multivariate models were developed on one part of the data (90 %) and validated on the independent part (10 %). The advantage of K-fold cross validation is that all the cases in the dataset are consecutively used for both model development and validation. The average performance of the models was calculated using AUC. Stepwise backward selection of variables was applied in every training sample [22, 23].

## Results

There were 11,407 employees from 25 organisations who completed the HRA during the study period. A total of 1653 participants (14.5 %) met one or more exclusion criteria. Baseline characteristics of the 9784 included participants are described in Table 1. In total, 4.3 % of men and 0.2 % of women had a SCORE estimated CVD risk ≥5 %. Because the number of women with a SCORE ≥5 % was too low to produce a model of valid statistical inference (*n* = 8), we proceeded to develop a prediction model for men [24].

**Table 1:** Baseline characteristics of study sample (*n* = 9784)

	Men (*n* = 6189)		Women (*n* = 3565)	
Age (SD)	49.4	6.0	47.1	5.5
Education^a^				
Low (%)	973	15.7	1030	28.9
Middle (%)	1988	32.1	1430	40.1
High (%)	3228	52.2	1105	31.0
Ethnicity				
Caucasian (%)	5821	94.1	3187	89.4
Other (%)	368	5.9	378	10.6
Tobacco use				
None (%)	5294	85.5	2977	83.5
At least once a week (%)	469	7.6	251	7.0
At least 10 g per day (%)	426	6.9	337	9.5
Body mass index (SD)	25.7	3.2	24.7	4.1
BMI <25 (%)	2738	44.2	2202	61.8
Overweight: BMI ≥25 - <30 (%)	2923	47.2	988	27.7
Obese: BMI ≥30 (%)	528	8.5	375	10.5
Serum total cholesterol in mmol/l (SD)	5.8	1.0	5.5	1.0
History of hypercholesterolemia (%)	179	2.9	58	1.6
Systolic blood pressure in mmHg (SD)	135.9	16.2	126.9	17.0
History of hypertension (%)	207	3.3	155	4.3
History of diabetes mellitus (%)	19	0.3	13	0.4
SCORE-low risk 5–10 (%)	235	3.8	7	0.1
SCORE-low risk >10 (%)	31	0.5	1	0.0
History of renal insufficiency (%)	72	1.2	29	0.8
eGFR (mL/min per 1.73 m^2^)				
≥ 90 (%)	3363	54.3	1822	51.1
60–90 (%)	2678	43.3	1612	45.2
45–59 (%)	143	2.3	127	3.6
30–44 (%)	5	0.1	4	0.1
< 30 (%)	0	0	0	0
Increased ACR (%)	109	1.8	70	2.0

^a^Education level. Low: lower general secondary/lower vocational. Middle: higher general secondary/pre-university/ intermediate vocational. High: Higher vocational/university/doctorate

eGFR: estimated glomerular filtration rate based on the chronic kidney disease epidemiology collaboration (CKD-EPI) formula. ACR: albumin/creatinine ratio in urine. Increased values defined as ≥3.5 for male and ≥2.5 for female persons

### Model development

In univariate analysis, 12 of the 23 selected variables were predictive of the SCORE estimated CVD risk ≥5 % threshold (Additional file 1: Table S1) and subsequently entered in the multivariate analysis. Table 2 shows the six variables retained in the final model. Age, tobacco use, self-reported history of hypertension (without current treatment), alcohol consumption, BMI and abdominal obesity independently predicted a ≥5 % SCORE risk. To facilitate practical use of the CVD risk score, β’s of these variables were multiplied and rounded to the nearest integer. A multiplication factor of 7 was chosen to sustain sufficient discriminative power between different predictor variables, resulting in a total CVD risk score ranging from 0 to 96.

**Table 2:** Multivariate regression of high cardiovascular disease risk in men for sociodemographic, lifestyle, and biometric variables (*n* = 6189)

		SCORE risk ≥5 %^a^				
			95.0 % C.I.			
		Odds Ratio	Lower	Upper	β	Risk Score^b^
Age	40–49^c^					0
	50–54	15.517	4.644	51.844	2.742	19
	55–59	206.816	64.758	660.507	5.332	37
	60–70	1168.532	354.895	3847.520	7.064	49
Tobacco use	None^c^					0
	At least once a week	14.232	9.977	20.300	2.655	19
Alcohol	<1 units per week^c^					0
Consumption	1–7 units per week	1.142	.688	1.895	.132	1
	8–14 units per week	1.278	.753	2.170	.246	2
	15–21 units per week	2.035	1.135	3.648	.710	5
	≥22 units per week	2.376	1.295	4.360	.866	6
Body mass index	Normal weight: BMI <25 kg/m^2c^					0
(BMI)	Overweight: BMI ≥ 25 - <30 kg/m^2^	1.687	1.130	2.520	.523	4
	Obese: BMI ≥30 kg/m^2^	1.932	1.043	3.579	.659	5
Waist circumference	<94 cm					0
	≥94 cm	1.849	1.238	2.760	.615	4
History of hypertension	No					0
	Yes	6.158	3.551	10.680	1.818	13

^a^Based on the SCORE equation for countries with low cardiovascular risk

^b^The risk score is produced by multiplying β’s by 7 and rounding them to the nearest integer

^c^indicates reference category

### Model validation

Ten-fold cross-validation resulted in an AUC of 0.95 (95 % CI [0.94–0.95]), demonstrating good discriminatory power. Diagnostic classification accuracy of the risk score at multiple cut-off values is shown in Table 3. To illustrate the influence of individual parameters on the outcome of the model several case examples are depicted in Table 4 using a cut-off value of ≥45. At this cut-off, 18 % of the study population has an estimated CVD risk ≥5 %.

**Table 3:** Diagnostic classification accuracy of predicting high CVD risk at different cut-off values

	TP	FN	FP	TN	Sensitivity	Specificity	PPV	LR +	LR-
Cut-off ≥40	254	12	1181	4742	95.5 %	80.1 %	17.7 %	4.8	0.1
Cut-off ≥45	247	19	888	5035	92.9 %	85.0 %	21.8 %	6.2	0.1
Cut-off ≥50	198	68	438	5485	74.4 %	92.6 %	31.1 %	10.1	0.3

*CVD* cardiovascular disease, *TP* true positive, *FN* false negative, *FP* false positive, *TN* true negative, *PPV* positive predictive value, *LR*+ positive likelihood ratio, *LR*- negative likelihood ratio

**Table 4:** Case examples of the cardiovascular disease risk screening tool using a cut-off of ≥45 points

Example nr.	Age	Tobacco use	Alcohol consumption	BMI	Waist circumference	History of hypertension	Risk Score	Estimated SCORE ≥5 %?^a^
1	52	No	8–14	≥25– <30	<94 cm	No	25	No
2	52	Yes	15–21	≥25– <30	≥94 cm	No	51	Yes
3	57	No	8–14	≥25– <30	<94 cm	No	43	No
4	57	Yes	<1	<25	<94 cm	No	56	Yes
5	47	Yes	15–21	≥30	≥94 cm	No	33	No
6	57	No	1–7	<25	<94 cm	Yes	51	Yes
7	62	No	<1	<25	<94 cm	No	49	Yes

*BMI* body mass index

^a^based on a cut-off of ≥45 points

## Discussion

We developed and validated a simple six-item CVD risk score that can be used as a first step in identifying male employees at high CVD risk based on current European guidelines using the SCORE risk estimation. At a cut-off of ≥45, only 18 % of screened persons where qualified as high CVD risk requiring further risk assessment, with a false-negative rate of 7 %. Because of the low prevalence of women with increased cardiovascular risk before age 65, screening women for CVD in the context of a worksite HRA does not seem to be efficacious.

Our proposed CVD risk score is developed to facilitate large scale CVD screening programmes based on the current guidelines by offering an easy and highly accurate first step in the screening process [4]. Instead of applying full CVD screening with BP measurement and blood sampling to all men aged 40 years and above, the CVD risk score can be used to preselect persons who require a total risk estimation. Applying this stepped approach means that BP and cholesterol measurement is required in only a small fraction of the screened population. The CVD risk score therefore seems a useful tool in reducing the costs, means and time needed to perform large scale screening. Choosing the cut-off value on the CVD risk score is a matter of policy and dependent on the acceptable percentage of false negatives and false positives in a particular screening setting. The high accuracy of our screening tool, however, seems to provide an acceptable trade-off between both rates.

Although we emphasize that ideally – as recommended in current guidelines - a full risk estimation is performed in every eligible person, most practices lack the time or finances to do so [7]. In such situations our CVD risk score can be employed instead as a first step in the risk assessment. A possible pitfall of using the CVD risk score compared to the original SCORE risk estimate, is that individuals who are at increased CVD risk because of isolated highly elevated BP or cholesterol could be missed as both are not measured. Other important cardiovascular risk factors such as diabetes or chronic kidney disease are not included in the SCORE formula, and persons with these risk factors could also be missed. However, this only applies to persons with risk factors that are untreated, as we excluded persons on current treatment for hypertension, hypercholesterolemia, diabetes, or chronic kidney disease because they are already at increased CVD risk. The number of persons with untreated risk factors is likely to be small in a relatively healthy working population. This is supported by the fact that in our population the number of subjects with moderate to severe renal function (eGFR < 45 ml/min/1.73 m^2^) was only 0.1 %. Nonetheless, because the proposed screening strategy is based on the identification of patients at risk for CVD with a simple questionnaire persons with these risk factors could remain unidentified. This should be taken in consideration when using the CVD risk score.

The performance of the original SCORE risk ranges from reasonable to good (AUC 0.71–0.84) [5]. Our CVD risk score is likely to resemble this performance, given its high accuracy in predicting the 10-year mortality risk of ≥5 %. Age and tobacco conferred the largest predictive value in our proposed risk score which is not surprising as they are both included in the SCORE risk assessment. Next to these variables, alcohol consumption, BMI, waist circumference and a history of hypertension (but currently untreated) independently predicted ≥5 % SCORE risk. It is likely that they act as a surrogate for the remaining SCORE variables, systolic BP and total cholesterol. High BMI and a large waist circumference often coincide with a high BP or dyslipidemia as part of the metabolic syndrome [25]. A history of hypertension indicates that the person has or is prone to develop hypertension. Although alcohol consumption might even be protective for development of the metabolic syndrome [26], there is a positive correlation between alcohol consumption and increased BP [27]. The contribution to the CVD risk score of these four variables is smaller than age and tobacco, but still these variables can be decisive in determining the screening outcome. In addition, these variables can also be used for a tailored lifestyle advice.

The low prevalence of women that reached the ≥5 % SCORE threshold in the current study population is in line with findings of a previous study comprising two Dutch population cohorts of similar age as the current population [28], where 0.1 % of the women and 3.1 % of the men reached the ≥5 % SCORE threshold. These findings are not surprising given that the ≥5 % threshold for low risk countries is not reached for non-smoking women until the age of 65, or 60 for smoking women, irrespective of BP or cholesterol [5]. Based on these numbers screening for CVD in women aged <60 years seems not useful from a worksite health care perspective in low-risk countries.

There are several limitations to our study that need further consideration. First, the proposed CVD risk score is developed and validated in a cohort of employees, which possibly limits the external validity of the screening tool when used in the general population. Nonetheless, the workplace provides an ideal setting for CVD risk screening as most men in the targeted age range (40–70) are part of the working population, and because it can facilitate the creation of a health-conscious environment [29]. Second, we did not include sedentary lifestyle in the questionnaire of our HRA. Sedentary lifestyle is one of the major risk factors for CVD [30], and inclusion of this non-invasive parameter to our questionnaire could have further increased the accuracy of our CVD risk score. Third, we have no data on CVD outcome in our population. As the aim of the study was to build a model to identify subjects with high SCORE risk, in line with current guideline recommendations, these data were not required. It would be, however, interesting in future research to validate the model on actual CVD outcome. F, the proposed CVD risk score can only be used in countries with low CVD population risk. However, the methods described in the current study can also be used to develop a similar model for high-risk countries. Finally, the proposed CVD risk score includes self-reported length and weight, which could lead to a slight underestimation of the calculated BMI [31].

## Conclusion

In conclusion, we used the data of a health risk assessment conducted in 25 Dutch organisations to construct a proposal for a simple six-item CVD risk score to identify individuals at increased CVD risk as defined by the SCORE risk estimation. The present risk score can be offered online as a simple, quick and inexpensive first step in the identification of persons at high cardiovascular risk, who subsequently qualify for further risk profiling according to the SCORE formula, including BP and cholesterol measures. We designed and validated our tool in population of workers from a European country at low CVD risk. Future studies should investigate whether the newly developed risk score can also be applied for other populations. Studies implementing our screening tool are warranted to evaluate the cost-effectiveness of a stepped approach for CVD risk screening as part of primary prevention strategies.

## Additional file

Additional file 1: Table S1. Univariate regression of high cardiovascular disease risk in men for sociodemographic, lifestyle and biometric variables. (DOC 106 kb)
