# Supplementary material for: A six question screen to facilitate primary cardiovascular disease prevention
Source: BMC Cardiovasc Disord. 2015 Oct 30;15:140. doi: 10.1186/s12872-015-0131-0 (PMC4628315; doi:10.1186/s12872-015-0131-0)
Supplement: Additional file 1: Table S1. — Univariate regression of high cardiovascular disease risk in men for sociodemographic, lifestyle and biometric variables. (DOC 106 kb) [file 12872_2015_131_MOESM1_ESM.doc]

| **Supplementary Table 1. Univariate regression of high cardiovascular disease risk in men for sociodemographic, lifestyle and biometric variables** | | |  |  |  |  |  |  |
| --- | --- | --- | --- | --- | --- | --- | --- | --- |
|  |  |  |  |  | **SCORE risk ≥ 5%^§^** | | |  |
|  |  |  |  |  |  | 95.0% C.I. | |  |
|  |  | *n* | *%* | B | Odds | Lower | Upper | Significant at *p*<0.10 |
|  |  |  |  |  |  |  |  |  |
| **Age** | 40-49 ^¥^ | 3232 | 52.2% |  |  |  |  | * |
|  | 50-54 | 1560 | 25.2% | 2.864 | 17.530 | 5.285 | 58.149 |  |
|  | 55-59 | 1085 | 17.5% | 5.022 | 151.660 | 48.191 | 477.282 |  |
|  | 60-70 | 312 | 5.0% | 6.288 | 538.167 | 169.329 | 1710.414 |  |
|  |  |  |  |  |  |  |  |  |
| **Education^†^** | Low ^¥^ | 973 | 15.7% |  |  |  |  | * |
|  | Middle | 1988 | 32.1% | -.532 | .588 | .429 | .805 |  |
|  | High | 3228 | 52.2% | -.981 | .375 | .275 | .511 |  |
|  |  |  |  |  |  |  |  |  |
| **Marital Status** | Maried/registered partner ^¥^ | 4884 | 78.9% |  |  |  |  | * |
|  | Divorced | 332 | 5.4% | -.343 | .710 | .383 | 1.313 |  |
|  | Cohabitant | 628 | 10.1% | -.680 | .507 | .298 | .860 |  |
|  | Widowed | 33 | 0.5% | 1.718 | 5.575 | 2.394 | 12.981 |  |
|  | Single | 288 | 4.7% | -.662 | .516 | .241 | 1.105 |  |
|  | Other | 24 | 0.4% | -.105 | .900 | .121 | 6.696 |  |
|  |  |  |  |  |  |  |  |  |
| **Ethnicity** | Caucasian ^¥^ | 5821 |  |  |  |  |  |  |
|  | Other | 368 | 5.9% | -.397 | .673 | .364 | 1.241 |  |
|  |  |  |  |  |  |  |  |  |
| **Self-rated health** | Very good ^¥^ | 1277 | 20.6% |  |  |  |  | * |
|  | Good | 4161 | 67.2% | .403 | 1.496 | 1.058 | 2.116 |  |
|  | Not good and not bad | 711 | 11.5% | .377 | 1.457 | .907 | 2.342 |  |
|  | Poor or very poor | 40 | 0.6% | .487 | 1.628 | .379 | 6.983 |  |
|  |  |  |  |  |  |  |  |  |
| **Tobacco use** | None ^¥^ | 5294 | 85.5% |  |  |  |  | * |
|  | At least once a week | 895 | 14.5% | 1.731 | 5.644 | 4.384 | 7.268 |  |
|  |  |  |  |  |  |  |  |  |
| **Alcohol** | <1 units per week ^¥^ | 1100 | 17.8% |  |  |  |  | * |
| **consumption** | 1-7 units per week | 2577 | 41.6% | .255 | 1.290 | .842 | 1.976 |  |
|  | 8-14 units per week | 1433 | 23.2% | .655 | 1.925 | 1.241 | 2.987 |  |
|  | 15-21 units per week | 664 | 10.7% | .914 | 2.494 | 1.538 | 4.044 |  |
|  | ≥22 units per week | 415 | 6.7% | 1.285 | 3.615 | 2.192 | 5.960 |  |
|  |  |  |  |  |  |  |  |  |
| **Nutrition** | Low vegetable/fruits intake | 5561 | 89.9% | -.238 | .788 | .542 | 1.146 |  |
|  | High saturated fat intake | 3980 | 64.3% | -.395 | .674 | .526 | .863 | * |
|  | Fish consumption <1 x per week | 2367 | 38.2% | -.217 | .805 | .620 | 1.043 |  |
|  |  |  |  |  |  |  |  |  |
| **Stress at work** | Never ^¥^ | 967 | 15.6% |  |  |  |  | * |
| **or home** | Some periods | 3392 | 54.8% | -.579 | .561 | .416 | .757 |  |
|  | Several periods | 1756 | 28.4% | -.869 | .419 | .291 | .605 |  |
|  | Permanent | 74 | 1.2% | .154 | 1.167 | .489 | 2.785 |  |
|  |  |  |  |  |  |  |  |  |
| **Distress** |  | 675 | 5.7% | .116 | 1.123 | .769 | 1.639 |  |
|  |  |  |  |  |  |  |  |  |
| **Current psychological treatment** | | 175 | 1.5% | .194 | 1.215 | .614 | 2.403 |  |
|  |  |  |  |  |  |  |  |  |
| **1st degree** | Diabetes Mellitus | 1159 | 18.7% | -.021 | .979 | .713 | 1.344 |  |
| **family history** | Hypertension | 2295 | 37.1% | -.131 | .877 | .677 | 1.137 |  |
|  | Cardiovascular disease | 656 | 10.6% | .112 | 1.118 | .762 | 1.641 |  |
|  |  |  |  |  |  |  |  |  |
| **History of** | Diabetes Mellitus | 19 | 0.3% | .213 | 1.238 | .165 | 9.308 |  |
|  | Hypertension | 207 | 3.3% | 1.282 | 3.604 | 2.357 | 5.512 | * |
|  | Hypercholesterolemia | 179 | 2.9% | .955 | 2.598 | 1.570 | 4.297 | * |
|  | Renal insufficiency | 72 | 1.2% | .515 | 1.674 | .669 | 4.189 |  |
|  |  |  |  |  |  |  |  |  |
| **Exercise, days per week ≥30 min.(0-7)** | |  |  | .000 | 1.000 | .998 | 1.002 |  |
|  |  |  |  |  |  |  |  |  |
| **Body mass index** | Normal weight:BMI <25 kg/m² ^¥^ | 2738 | 44.2% |  |  |  |  | * |
| **(BMI)** | Overweight: BMI ≥25 - <30 kg/m² | 2923 | 47.2% | .822 | 2.276 | 1.716 | 3.020 |  |
|  | Obese: BMI ≥30 kg/m² | 528 | 8.5% | .744 | 2.104 | 1.344 | 3.291 |  |
|  |  |  |  |  |  |  |  |  |
| **Waist** | <94 cm ^¥^ | 2828 | 45.7% |  |  |  |  | * |
| **circumference** | ≥94 cm | 3361 | 54.3% | .953 | 2.593 | 1.957 | 3.437 |  |

**^§^**Based on the SCORE equation for countries with low cardiovascular risk. ^¥^indicates reference category. **^†^**Education level. Low: lower general secondary/lower vocational. Middle: higher general secondary/pre-university/ intermediate vocational. High: Higher vocational/university/doctorate.
